# The pathological features of ectopic lymphoid neogenesis in idiopathic dacryoadenitis

**DOI:** 10.1186/s12886-016-0250-0

**Published:** 2016-05-26

**Authors:** Jie Guo, Jiang Qian, Rui Zhang

**Affiliations:** Department of Ophthalmology, Eye & ENT Hospital, Fudan University, Shanghai, China; Shanghai Key Laboratory of Visual Impairment and Restoration, Fudan University, Fenyang Road No. 83, Shanghai, 200031 China

**Keywords:** Idiopathic dacryoadenitis, Ectopic lymphoid neogenesis, Follicular dendritic cells, CXCL 13, CCL21

## Abstract

**Background:**

Lymphoid neogenesis has been reported in various diseases but not in idiopathic dacryoadenitis. The aim of this paper is to discuss the pathological features of lymphoid neogenesis in idiopathic dacryoadenitis.

**Methods:**

20 cases of idiopathic dacryoadenitis were collected retrospectively. Lymphoid neogenesis was graded by lymphocytic aggregates and germinal center-like structure formation. T and B cell compartmentalization, follicular dendritic cells and the expression of CXCL13 and CCL21 were analyzed.

**Results:**

Grade 1 lymphoid neogenesis was observed in 10 of 20 cases (50 %), grade 2 in 18 of 20 cases (90 %) and grade 3 in 14 of 20 (70 %). The existence of T and B cell compartmentalization and follicular dendritic cells increased in parallel to the grade of lymphoid neogenesis. The expression of CXCL13 significantly increased in the higher grade of lymphoid neogenesis, but no correlation was found between CCL21 and grades of lymphoid neogenesis.

**Conclusions:**

Ectopic lymphoid neogenesis participates in the pathogenesis of idiopathic dacryoadenitis and appears as a dynamic process.

## Background

Idiopathic dacryoadenitis, also known as a lacrimal gland pseudotumor, is inflammation of the lacrimal gland tissue with no identifiable local or systemic cause. Idiopathic dacryoadenitis has been considered an immune-mediated process. It is a very common type of orbital pseudotumor, accounting for approximately 20 %–57 % of cases [1–3]. It is also the usual cause of bilateral lacrimal gland disease [4].

Lymphoid infiltration is a typical pathological feature of idiopathic dacryoadenitis. It may destroy normal tissue by the formation of T cell-B cell follicles with germinal center (GC) reactions, which were defined as ectopic lymphoid neogenesis. Ectopic lymphoid neogenesis has been observed in many autoimmune diseases and chronic inflammation, such as rheumatoid arthritis [5], Sjögren’s syndrome [6], psoriatic arthritis [7], Hashimoto thyroiditis [8], etc. Based on our observation of the pathological change of idiopathic dacryoadenitis, we suggest that lymphoid neogenesis may also play an important role in this disease.

Lymphoid neogenesis in chronic inflammatory disease is a complex process regulated by an array of cytokines, chemokines and adhesion molecules [9]. Among them, the B cell–attracting chemokine, CXCL13, required for the normal polarization of GCs, was studied most widely and has been implicated as a key regulator of lymphoid neogenesis in many diseases [10]. Additionally, CCL21, as a chemoattractant for T cells and dendritic cells, was also researched widely in previous studies [11].

In this study, we discuss the pathological feature of ectopic lymphoid neogenesis in idiopathic dacryoadenitis, as well as the expression of follicular dendritic cells (FDCs), CXCL 13 and CCL21.

## Methods

### Patient collection

20 cases of idiopathic dacryoadenitis were collected retrospectively from Shanghai Eye and ENT Hospital of Fudan University from 2009 to 2014; all the specimens came from therapeutic surgical excision or biopsy. The diagnoses were based on the history of clinical presentations, symptoms, examinations and pathological findings, and ruled out foreign body reaction, inflammation with an infectious agent, sarcoidosis, Wegener’s granulomatosis, Sjögren syndrome, Mikulicz’s disease, lymphoid hyperplasia and lymphoma. Two pathologists determined the diagnosis by mutual agreement. Medical records were retrospectively obtained, including age, gender and disease duration.

The study adhered to the tenets of the Declaration of Helsinki. The study was approved by the Ethics Committee of the Eye and ENT Hospital of Fudan University.

### Histology

**Fig. 1:**
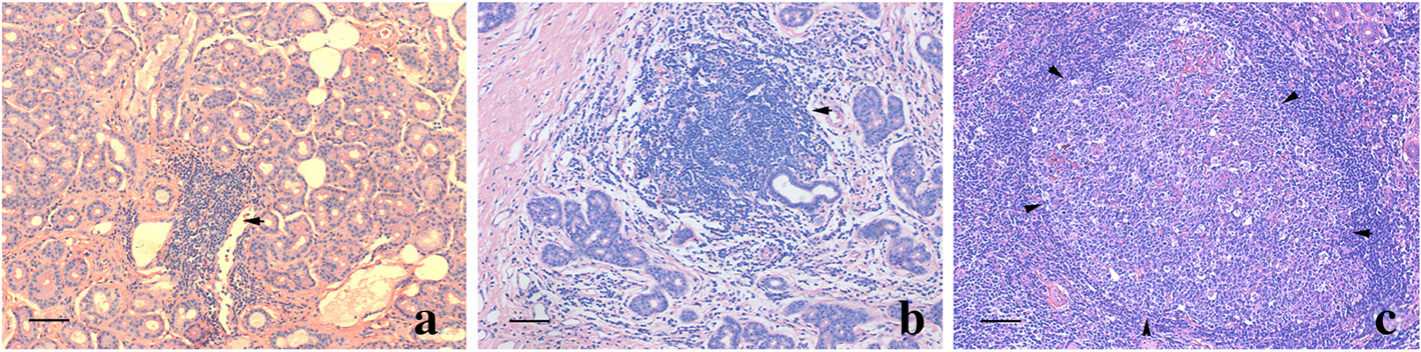
The grades of lymphoid neogenesis in idiopathic dacryoadenitis. **a** Grade 1 was defined as lymphocytic aggregates corresponding to 10–20 radial cell counts (arrow). **b** Grade 2 was defined as more than 20 radial cell aggregations but without GC-like structure formation (arrow). **c** Grade 3 was defined as massive lymphocytic aggregates with GC-like structure formation (arrows indicate the localization of GC-like structure). Scale bar = 100 μm

Hematoxylin sections of lacrimal gland tissue samples were analyzed for the organization of lymphocytic infiltration. Lymphoid neogenesis has been assessed by different criteria in previous reports. Based on our observation of the samples, we graded lymphoid neogenesis by lymphocytic aggregates and GC-like structure formation. Briefly, a mass of lymphocytic aggregates and its approximate center were defined, then the radial line was draw from the center to the margin of the aggregates, and the number of cells through this line was counted: grade 1 lymphocytic aggregates corresponded to 10–20 radial cell counts; grade 2 ≥ 20 radial cell counts without GC-like structure formation; and grade 3 lymphocytic aggregates were defined as massive lymphocytes with GC-like structures formation (Fig. 1). Three sections from different parts of the sample were analyzed for each case, and the one with the most obvious lymphocytic aggregates was chosen. GC-like structures within lymphoid follicles were identified as follows: included well-circumscribed clusters of centrocytes and centroblasts with variable numbers of tingible body macrophages and mitotic figures within aggregates of small lymphocytes.

### Immunohistochemistry

Immunohistochemical staining was performed on tissue sections using monoclonal antibodies against CD20 (10 μg/ml, BM0442, Boster Bio-engineering), CD3 (10 μg/ml, BM0210, Boster Bio-engineering), CD35 (10 μg/ml, M0846, DakoCytomation), CXCL13 (10 μg/ml, AF801, R&D system) and CCL21 (10 μg/ml, AF366, R&D system). Sections were retrieved by boiling in a citrate buffer (pH 6.1) in a microwave (800 W) for 2 min before staining. Negative controls included non-immune-matched immunoglobulins instead of the primary antibodies.

The staining of the CD20 and CD3 was studied on sequential sections, and the topographical arrangement of T and B cells was analyzed: those displaying a trend toward organization into separate T cell–rich and B cell–rich areas were defined as T/B cell compartmentalization.

### Statistics

All values were expressed as mean and standard deviation (SD). The Mann–Whitney test was used for statistical analysis of nonparametric data. Chi-square analysis was employed for comparing the expression of CD35+ FDCs, CXCL13, CCL21 and T/B cell compartmentalization in different grades of lymphocytic aggregates. *P*-values <0.05 were considered statistically significant.

## Results

### Clinical features

The average age of patients was 48.7 ± 17.3 years (ranging from 23 to 79 years), including 10 males and 10 females. The cases included 11 right lacrimal glands and nine left. The disease durations were from one to 84 months (average 17.9 ± 20.5 months). The main ophthalmologic symptoms included swelling, proptosis, and redness, and a few with mild displacement of the eyeball. All the symptoms obviously improved after surgery, which was consistent with previous reports [12]. There was no statistical correlation between grades of lymphoid neogenesis and disease duration (*p* = 0.298). The demographic and clinical data of patients are shown in Table 1.

**Table 1:** The demographic and clinical data of patients

No	Gender	Age (years)	Disease duration (months)	Eye	Highest grade	Grade and T/B compartmentalization			CD35	CXCL13	CCL21
						Grade 3	Grade 2	Grade 1			
1	F	38	36	right	3	Yes	Yes	N/A	+	+	+
2	F	57	24	left	3	Yes	Yes	N/A	+	+	-
3	F	33	24	left	3	Yes	Yes	No	+	+	+
4	F	50	9	left	3	Yes	No	No	+	+	-
5	M	45	24	left	2	N/A	Yes	No	+	-	+
6	M	73	12	right	3	Yes	Yes	N/A	+	+	-
7	F	27	6	right	3	Yes	Yes	No	+	+	+
8	M	64	8	left	3	Yes	Yes	N/A	+	+	+
9	M	70	12	left	3	Yes	Yes	N/A	+	+	+
10	M	56	4	right	3	Yes	Yes	N/A	+	+	-
11	M	23	2	left	3	Yes	No	No	+	+	+
12	F	71	36	right	3	Yes	Yes	No	+	+	-
13	M	41	8	right	3	Yes	Yes	N/A	+	+	-
14	F	46	5	right	2	N/A	No	N/A	-	+	-
15	F	33	2	right	2	N/A	No	N/A	-	-	+
16	M	28	10	right	2	N/A	No	No	-	-	+
17	M	52	48	left	3	Yes	No	No	+	+	-
18	F	79	84	right	3	Yes	N/A	N/A	+	+	+
19	M	60	1	right	2	N/A	No	No	-	-	-
20	F	27	3	left	1	N/A	N/A	No	-	-	-

### Histomorphologic grading

Inflammatory cell infiltration was a typical finding in lacrimal gland lesions, mostly in lymphocytes with some polymorph nuclear leucocytes, plasma cells, macrophages and eosinophils. Fibrosis was variable, and abundant lymphocytic aggregates with little fibrosis were observed in most cases, whereas in some cases, small lymphocytic aggregates localized discretely in dense fibrosis.

Different degrees of lymphocytic aggregates were found in these cases. Grade 1 aggregates were observed in 10 of 20 patients (50 %), grade 2 in 18 of 20 patients (90 %) and grade 3 in 14 patients (70 %) (Fig. 1). The highest grade of each case was also recorded to reflect the severity of lymphoid pathological change: only one case (5 %) had only grade 1 change, 5 cases (25 %) had the highest change of grade 2, and 14 (70 %) were grade 3.

**Fig. 2:**
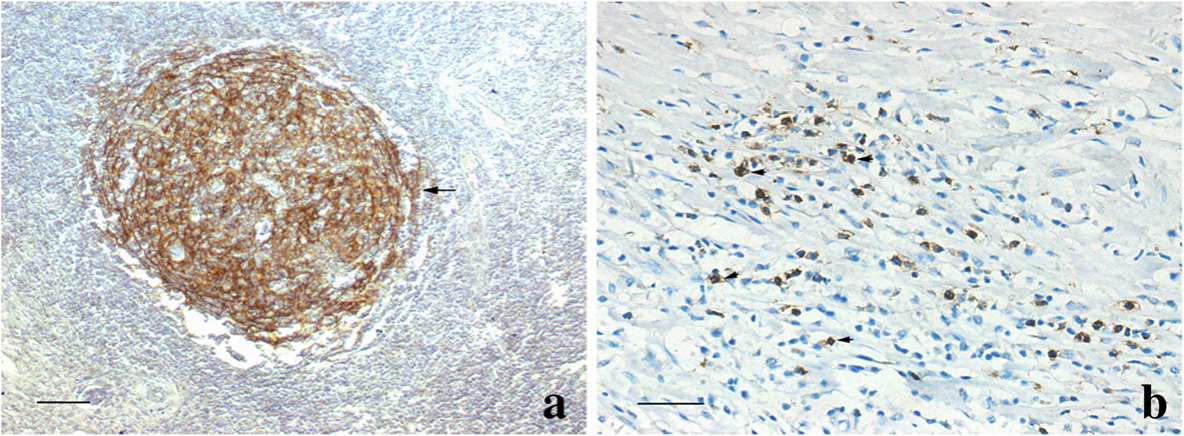
The expression of CD35+ cells in lymphoid neogenesis. **a** The network of CD35+ FDCs in GC-like structures (grade 3) (arrow). Scale bar = 100 μm (**b**) In some grade 2 areas, CD35+ cells were scattered within the lymphoid aggregation areas (arrows). Scale bar = 50 μm

Additionally, the expression of CD35+ FDCs was studied. CD35+ staining was detected in 15 of 20 cases (75 %): 14 cases presented as a network of FDCs in the light zone of GC-like structures (grade 3 areas), among which six cases also showed scattered CD35+ cells in grade 2 lymphocytic aggregates without network formation (Fig. 2); one case had grade 2 lymphocytic aggregates only, and CD35+ cells were detected scattered in lymphocytic aggregates areas. CD35+ staining was not found in any grade 1 areas. The CD35+ FDC expression was significantly different in the three grades of lymphoid neogenesis (*p* = 0.002).

### T and B cells compartmentalization

**Fig. 3:**
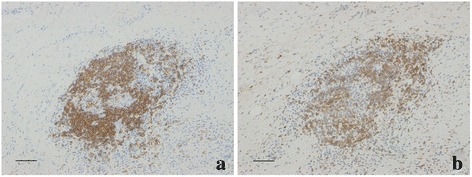
T and B cell compartmentalization in grade 2 lymphocytic aggregates. **a** and **b** showed B and T lymphocytes respectively. Scale bar = 100 μm

**Fig. 4:**
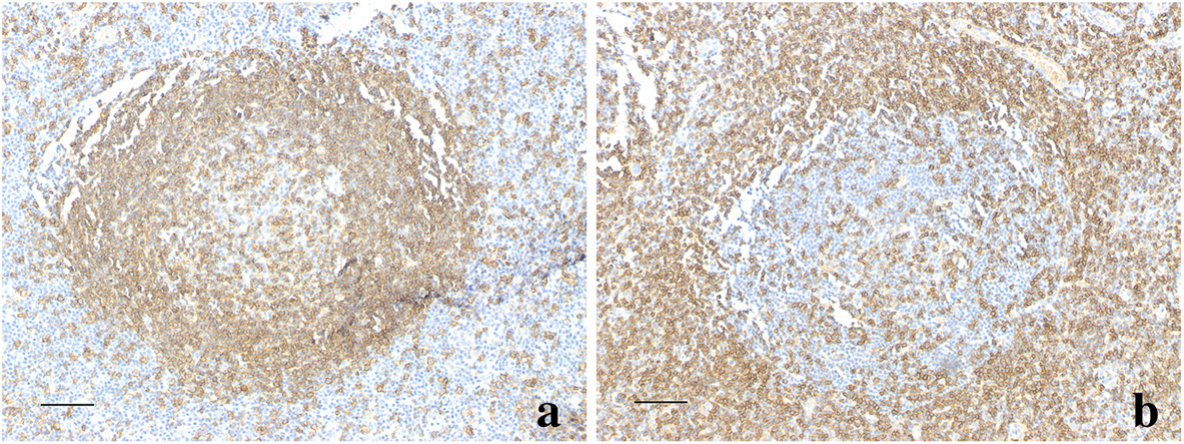
The grade 3 lymphoid neogenesis displayed a typical segregation of T and B cells in GC-like structures. **a** and **b** showed B and T lymphocytes respectively. Scale bar = 100 μm

T and B cell compartmentalization increased in parallel to the grades of lymphoid neogenesis. None of the grade 1 areas showed evidence of defined T/B cell compartmentalization. In 18 cases with grade 2 lymphocytic aggregates, 11 cases (61.1 %) existed of T/B cell compartmentalization in grade 2 areas (Fig. 3). All of the grade 3 lymphocytic aggregate areas (100 %) displayed a typical segregation of T and B cells, surrounding a central area consisting of GC-like structures (Fig. 4). There was a significant difference of proportion of T/B cell compartmentalization in different grades (*p* < 0.001).

### Expression of CXCL13 and CCL21

**Fig. 5:**
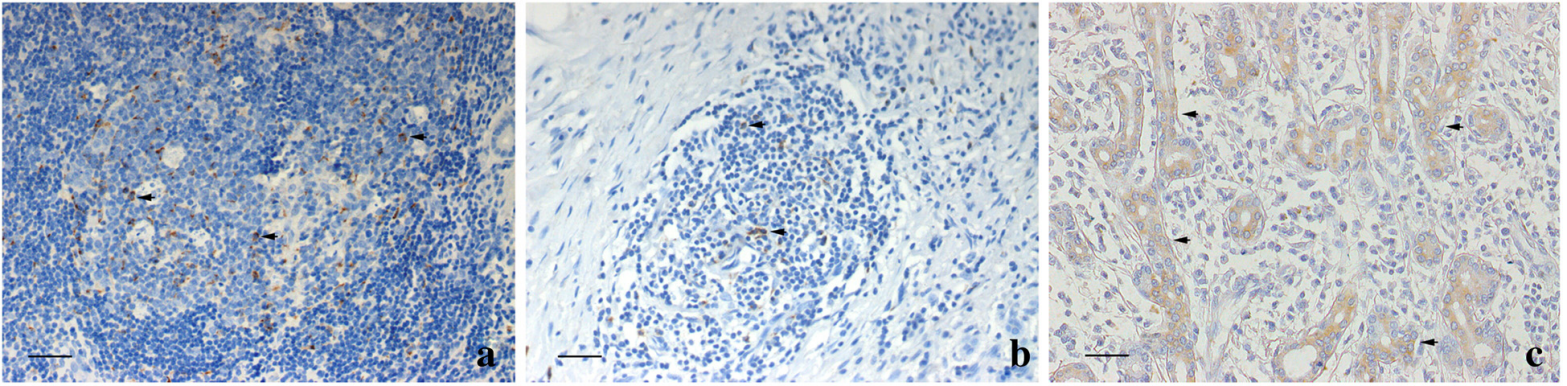
The expression of CXCL 13 in different grades of lymphoid neogenesis. **a** In grade 3, CXCL13 was expressed in GC-like structures of lymphoid follicles (arrows). **b** In grade 2 areas, CXCL13 was mostly scattered in the center of lymphoid aggregation (arrows). **c** CCL21 was expressed by acinar and ductal epithelial cells (arrows). Scale bar = 50 μm

CXCL13 was expressed by mononuclear cells. In grade 3 lymphoid neogenesis areas, CXCL13 preferentially presented within GC-like areas of lymphoid follicles and few were scattered in the surrounding areas (Fig. 5a); in grade 2 areas, we found CXCL13 mostly scattered in the center of lymphoid foci (Fig. 5b). Positive staining for CXCL13 was observed in 15 of 20 cases (75 %). Additionally, we compared the expression of CXCL13 in different grades of lymphoid neogenesis respectively: CXCL13+ staining within grade 1 areas was observed only in one case (10 % of the cases with grade 1 lymphoid neogenesis). In grade 2 areas it increased to seven cases (38.9 % of the cases with grade 2 lymphoid neogenesis), and for grade 3 areas CXCL13+ staining existed in all 14 grade 3 cases (100 %). The expression of CXCL13 was significantly different in different grades (*p* = 0.001).

CCL21 was expressed by acinar and ductal epithelial cells (Fig. 5c), and positive staining was observed in 10 of 20 (50 %) cases. We also compared the expression of CCL21 in different grades of lymphoid neogenesis respectively. However, no correlation between CCL21 and grades of lymphoid neogenesis was found (*p* = 0.7).

## Discussion

Lymphoid infiltration is the main process in most immune-mediated diseases. Emergence of GC follicles in extranodal sites is considered a critical step in the generation of the autoimmune process. Ectopic lymphoid neogenesis has been well studied in various chronic inflammatory diseases such as Sjögren’s syndrome, autoimmune thyroid disease, rheumatoid arthritis and chronic infection [13, 14]. However, the features of their lymphoid organizations range from simple aggregates of B cells and T cells to highly ordered follicle structures, so the criteria of lymphoid neogenesis vary throughout the existing research [5, 15, 16]. These differences may depend on varying immune responses and disease characteristics, including response between antigens and antibodies, cytokines, chemokines, and other influencing factors. In idiopathic dacryoadenitis, normal tissue is replaced by intensive lymphocyte infiltration, and lymphoid follicles with GCs develop easily. Therefore, our criteria presented a larger aggregation of lymphocytes compared to previous reports, which we believed could better indicate the feature of lymphoid neogenesis in idiopathic dacryoadenitis. In our study, lymphoid neogenesis with GC-like structures was present in most (70 %) cases, the proportion is beyond most other diseases in previous reports (5 %–60 %) [5, 17–19].

It is widely believed that ectopic lymphoid neogenesis is a dynamic process. In most of our cases, different grades of lymphoid neogenesis existed simultaneously. The development of lymphocyte organization was parallel to the grades. Grade 1 lymphoid neogenesis showed lymphocytic aggregates only, more than half of grade 2 cases showed obvious T and B cell compartmentalization, and all grade 3 lesions had a typical follicle organization. FDCs are an essential cellular component of B-cell follicles in lymphoid neogenesis. The FDC network presented in all grade 3 areas and was scattered in some grade 2 areas, which indicates that lymphoid neogenesis in idiopathic dacryoadenitis may depend on an antigen recognition event.

The microstructure of GC-like structures in the lacrimal gland was similar to that in lymphoid tissues, which may suggest the immunological competence of these structures as GCs. GCs play a critical role in the development of B cell immune responses because they provide an infrastructure to capture and store the antigen, which drives B cell development and differentiation. The homeostatic trafficking of B cells into the lymphoid tissue and B cell follicles appears to be critically controlled by B lymphocyte chemoattractant CXCL13. In conformity with previous research, CXCL13 was locally expressed and closely associated with the process of lymphoid neogenesis in our study, although this correlation was not always proven in previous research [6]. CCL21 may also play a role in mediating the homing of lymphocytes to secondary lymphoid organs; it is a high-affinity functional ligand for chemokine receptor 7 that is expressed on T and B lymphocytes. Some research has reported that the expression of CCL21 increased in tissue containing ectopic GC reactions [5], but we could not confirm this in idiopathic dacryoadenitis; the function of CCL21 in this disease needs further discussion.

Additionally, our study found that ectopic lymphoid neogenesis in the lacrimal gland was not related to disease duration. There was no statistical significance between the severity of fibrosis and lymphoid neogenesis in idiopathic dacryoadenitis (data is not shown here).

## Conclusion

In conclusion, the study suggests that ectopic lymphoid neogenesis participates in the pathogenesis of idiopathic dacryoadenitis. Lymphoid neogenesis is a dynamic, consecutive process. Lymphocytic aggregates, T and B cell compartmentalization, and GC-like structure formation may be the primary steps. FDCs and CXCL13 may play an important role in this process.

## Abbreviations

FDC, follicular dendritic cell; GC, germinal centers; SD, standard deviation.
